# Dietary green-plant thylakoids decrease gastric emptying and gut transit, promote changes in the gut microbial flora, but does not cause steatorrhea

**DOI:** 10.1186/s12986-016-0128-4

**Published:** 2016-10-12

**Authors:** Eva-Lena Stenblom, Björn Weström, Caroline Linninge, Peter Bonn, Mary Farrell, Jens F. Rehfeld, Caroline Montelius

**Affiliations:** 1 Department of Experimental Medical Science, Appetite Regulation Unit, BMC B11, Lund University, Sölvegatan 19, SE-221 84 Lund, Sweden; 2 Department of Biology, Lund University, Sölvegatan 35, SE-223 62 Lund, Sweden; 3 Department of Food Technology, Engineering and Nutrition, Lund University, Box 124, SE-221 00 Lund, Sweden; 4 Medicinal Chemistry CVMD, AstraZeneca R&D, Mölndal, Sweden; 5 Department of Clinical Biochemistry, Rigshospitalet, Copenhagen University, DK-2100 Copenhagen, Denmark

**Keywords:** Intestinal transit, Microbiota, Faecal fat, Spinach, Obesity

## Abstract

Green-plant thylakoids increase satiety by affecting appetite hormones such as ghrelin, cholecystokinin (CCK) and glucagon-like peptide-1 (GLP-1). The objective of this study was to investigate if thylakoids also affect gastrointestinal (GI) passage and microbial composition. To analyse the effects on GI passage, 16 rats were gavage-fed a control or thylakoid-supplemented high-fat diet (HFD) 30 min before receiving Evans blue. Another 16 rats were fed a control HFD or thylakoid HFD for two weeks prior to the intragastric challenge with Evans blue. The amount of Evans blue in the stomach and the distance of migration in the intestines after 30 min were used as a measurement of gastric emptying and intestinal transit. These were reduced by thylakoid supplementation in the acute study, and however not significantly also after the two-week diet study.

The second aim of the study was to investigate if thylakoid-supplementation affects the gut microbiota and amount of faecal fat in healthy human volunteers (*n* = 34) receiving thylakoid or placebo treatments for three months. Microbiota was analysed using 16S rRNA gene sequencing and qPCR, and faecal fat was extracted by dichloromethane. The total bacteria, and specifically the *Bacteriodes fragilis* group, were increased by thylakoid treatment versus placebo, while thylakoids did not cause steatorrhea. Dietary supplementation with thylakoids thus affects satiety both via appetite hormones and GI fullness, and affects the microbial composition without causing GI adverse effects such as steatorrhea. This suggests thylakoids as a novel agent in prevention and treatment of obesity.

## Background

Satiety is regulated by several physiological and psychological factors, including the taste and composition of the meal, gastric distension and emptying rate, transit time through the gastrointestinal (GI)-tract, digestion and absorption of nutrients, secretion of satiety hormones and suppression of hunger hormones, as well as the awareness of previous mealtime [1]. Recent strategies in the fight against obesity, overweight and metabolic diseases are diet and exercise recommendations, pharmacological treatments, and irreversible surgeries such as gastric bypass. Since obesity increases the risk for cardiovascular disease, non-alcoholic fatty-liver disease, diabetes, various cancers, arthritis and cognitive impairment [2], the economical cost of medical treatments are increasing [3, 4]. Today, the pharmaceutical agents present for treating obesity include drugs affecting or mimicking appetite regulating hormones, digestion and/or absorption of nutrients, or rewarding and food-seeking areas of the brain. The therapeutic agent Orlistat works by inhibiting lipolysis, with the result that about 30 % of triglycerides in the food are not being digested and absorbed, hence less energy is absorbed. The result is approximately 10 % body weight loss in one year, but with medium to severe gastrointestinal symptoms, such as steatorrhea [5].

Thylakoids are part of the chloroplast in all green leaves, as they are the sites for the photosynthesis. Thylakoids consist of various membrane-bound proteins, galactolipids, phospholipids and antioxidants such as chlorophyll, carotenoids, zeaxanthin and lutein [6]. Thylakoids extracted from spinach have previously been found to prolong lipolysis [7], decrease glucose-uptake over the intestinal wall and to create an extra temporary layer covering the mucosal side of the intestine [8]. In animal studies, thylakoid supplementation results in a decreased amount of body fat [9] and decreased food intake [10, 11]. In human intervention studies, thylakoids have been shown to promote satiety by affecting subjective fullness, hunger and urge for specific foods, as well as to enhance the release of the appetite-suppressant hormones cholecystokinin (CCK), glucagon-like peptide-1 (GLP-1) and leptin, and to suppress the hunger-hormone ghrelin [12–16]. Effects on glucose and insulin homeostasis after standardised meals, as well as in the fasting condition, have also been reported [12, 13, 15]. Moreover, thylakoid supplementation promotes body weight loss in humans; 6.3 % in three months [12].

Alterations of the microbial composition in the gut have in recent years been identified as important for development of various disorders, including obesity [17]. Investigating physiological effects of the various bacterial groups has been proposed to be more important than the precise taxonomical composition. The known functions of the most occurring bacteria in the gut can so far be divided in the metabolic (affecting digestion), the protective (protecting against pathogens) and the trophic (affecting epithelial cell proliferation and differentiation) [17]. The bacterial groups affecting metabolism work through enzymatic reactions cleaving glycoside linkages present in polysaccharides and dietary fibres, resulting in oligosaccharides and monosaccharides which are then fermented into short-chain fatty acids (SCFA), such as acetate, propionate and butyrate [17]. The SCFA are then absorbed into the bloodstream, and utilized as substrates for gluconeogenesis and lipogenesis [17]. Another function of the SCFAs is to act as ligand to free fatty acid receptors, which have been found to affect satiety and insulin sensitivity [2]. The metabolic effect of this activation is induced expression of the satiety hormones peptide tyrosine-tyrosine (PYY) and GLP-1, secreted from intestinal enterocytes, and leptin secreted from adipose tissue [2]. The result would hypothetically be an increased satiety. Gut microbiota is also involved in increasing the bioavailability of antioxidants in the food consumed. This is due to the bacteria hydrolysing esters, glycosides and polymers in the food matrix, thereby releasing polyphenolic compounds [18].

The aim of the present study was to investigate how thylakoid supplementation affects gastric emptying, intestinal transit and plasma concentrations of CCK, using two rat models; one measuring the immediate effects of a thylakoid high-fat diet by gavage, and one measuring the effects after two-weeks of thylakoid supplementation in the diet. The second aim was to measure the changes in microbial composition in faeces, as well as the amount of faecal fat, after three-months daily thylakoid supplementation in overweight women.

## Methods

### Animal experiments

The experiments on gastric emptying and intestinal transit were performed on Sprague-Dawley rats. The rats were bred (at the animal facility at the department of Biology, Lund University, Sweden) and kept under specific pathogen-free conditions with a controlled environment (21 ± 2 °C, 50 ± 10 % relative humidity, a 12 h light – 12 h dark cycle). At 6w of age rats were placed in individual cages with ad libitum access to a standard rat chow-diet (R36, Lantmännen, Sweden) to be acclimatised for one week before the experiments started. In total 30 rats were used, divided between two experiments; one acute study (14 rats) and one two-week treatment study (16 rats). During both studies a HFD (46 % fat, 18 % protein and 36 % carbohydrates) produced by Research Diets Inc. (NJ, USA) was used; one control and one containing 0.33 g thylakoids/gram feed (Appethyl, Greenleaf Medical, Stockholm, Sweden). The control-HFD and thylakoid-HFD were matched for caloric and nutritional content. In the acute study, 1.3 g of the control-HFD or thylakoid-HFD was given by gavage, while during the two-week experiment rats had ad libitum access to their respective feed. The studies were approved by the Lund University Ethical Review Committee for Animal Experiments (no. M107-13) and were conducted according to the European Communities regulations concerning protection of experimental animals.

#### Acute study; HFD via gavage

In the acute study, 1.3 g of control HFD or thylakoid HFD were homogenised with 4 ml drinking water to obtain a slurry. The rats were lightly anaesthetized with fluothane, and 2.5 mL of the HFD-slurry was fed via a stomach tube (control HFD *n* = 6, and thylakoid HFD *n* = 8). The thylakoid fed rats received in total 0.4 g thylakoids in the 2.5 mL slurry. After 30 min, the rats were again lightly anaesthetized with fluothane and given an intragastric bolus dose of 0.25 mL/100 g body weight of 25 mg/mL Evans blue solution containing 1 % methylcellulose to obtain a semi-liquid solution. Sixty minutes after the HFD bolus feeding (30 min after Evans blue), the rats were deeply anaesthetized with fluothane before a laparotomy was performed. Blood was collected from the heart into EDTA tubes, and the rats were sacrificed by exsanguination. Blood samples were centrifuged at 3000 × g for 10 min at 4 °C and the plasma was collected and stored in -80 °C until analysed. The stomach and intestine were then removed, and the intestine clamped with two sutures beneath the pylorus to prevent leakage of the Evans blue solution. The small intestine was separated from the stomach, by cutting between the strings, and also cut just before the ceacum. The total length of the small intestine, as well as the migration of Evans blue, was measured. The small intestine was then divided into five equally long segments using bulldog clamps. Both the intestinal segments and the stomach were flushed with 10 mL of the flush solution (0.1 M NaOH + 6 mmol/L NAC) each, cut open and placed in 15 mL tubes (50 mL for the stomach). Tubes were shaken vigorously for 60 s and incubated in room temperature for 60 min.

#### Two-week ad libitum food intake

In the two-week treatment study, rats were divided into two groups; one receiving ad libitum access to thylakoid HFD (*n* = 8) and one receiving ad libitum access to control HFD (*n* = 8) for 2 weeks. On the last day, the rats were lightly anaesthetized with fluothane and an intragastric bolus dose of Evans blue was given by gavage. Thirty minutes later the rats were deeply anaesthetized and the same procedure as described for the acute study was performed.

#### Analyses of gastric emptying and intestinal transit

Intestinal and stomach samples were centrifuged at 3000 × g for 30 min at 4 °C, after which 1 mL of each sample was transferred into Eppendorf tubes and centrifuged at 16 000 × g for 60 min at 4 °C. 100 μL of each sample was finally loaded onto a 96-well plate and absorbance was read at wavelength 565 nm (A565). The percentages of gastric emptying and Evans migration were calculated using equations 1 and 2 [19].

*Equation 1. The percentage of gastric emptying was calculated by the formula:*

$$ \left[\frac{\Sigma A565\left( intestine\ 1-5\right)}{\Sigma 565\ \left( stomach+ intestine\ 1-5\right)}\right]\times 100 $$

*Equation 2. The percentage of Evans migration was calculated by the formula:*

$$ \left[\frac{length\  of\  migrated\  EVANS(cm)}{total\  length\  of\  intestine\ (cm)}\right]\times 100 $$

### Human experiment

Faecal samples were collected from 34 overweight women (*n* = 18 in the thylakoid group, *n* = 16 in the control group) who had been enrolled in a 12-week diet and treatment intervention study previously described [12]. A blueberry drink, with or without thylakoid supplementation, was ingested every morning for 12 weeks. The blueberry drink contained 50 mL blueberry soup (Ekströms original, Procordia Food AB, Eslöv, Sweden) and 2.8 g rapeseed oil (Zeta, Di Luca & Di Luca AB, Stockholm, Sweden). In the thylakoid blueberry drink 5 g Appethyl (Greenleaf Medical, Stockholm, Sweden) was added. The caloric content was 209 kJ/50 kcal in the thylakoid drink and 188 kJ/45 kcal in the control drink. Exclusion criteria were diabetes, food allergies and intolerance, irritable bowel syndrome, recent use of antibiotics (within six months from inclusion), as well as being a vegetarian, smoker, pregnant or being on any weight-loss diet for the last three months.

During the 12-week intervention period subjects were instructed to consume three meals per day, and no snacks between meals, as well as to carry out 30 min of low-to-medium intense exercise every day. During the 12 week intervention all subjects decreased in body weight, and the thylakoid group decreased significantly more in body weight and in blood-lipid levels. More details describing the human trial, and the results can be found in the previous study [12].

In this study only 34 out of the original 36 women were included, due to incompliance in handing in faecal samples. The experiment was conducted according to the guidelines laid down in the Declaration of Helsinki and all procedures involving human subjects were approved by the Ethics Committee of Lund University (no. 2006/361). Written and verbal informed consent was obtained from all participants before the start of the experiment.

The faecal samples were collected by the participants in their home environment within two days before arriving at the clinic and put in −20 °C directly after collection. The participants had received sterile 50 ml tubes with a small spoon attached in the lid, specially designed for the purpose. At the clinic, the collected samples were thawed and divided in two; one part was kept in −80 °C until bacterial analyses were made and the other part was put in an open glass container and left to dry in 100 °C for 48 h before the analysis of fat-content was performed.

#### Analysis of fat content

The dry faecal material (~100 mg) was placed in a pre-weighed 4 ml glass vial and weighed. A magnetic bead together with 2 ml of dichloromethane (DCM) (Chromasolv, Sigma-Aldrich, Stockholm, Sweden) was added. The vial was caped and the mixture was stirred vigorously for two hours. After the mixture had sedimented, the clear DCM phase was removed and transferred into a plastic syringe (10 ml), fitted with a polyethylene frit (20 μm, 10 ml, Biotage, Uppsala, Sweden) and a syringe filter (1 μm, Acrodisc glass fiber, Pall, NY, USA). The organic filtrate was collected in a pre-weighed glass vessel. To the vial containing the residue faeces, 2 ml more of DCM was added and the mixture was stirred vigorously for another two hours. The DCM phase was transferred as described above. The extraction sequence was repeated one more time to achieve a total of three times. When executing the last extraction, all the solid material was transferred together with the DCM to the syringe. To ensure that as much of the organic solvent as possible was filtered into the glass vessel, a plunger was used to squeeze the solid material. The solvent was then removed by evaporation in a vacuum centrifuge (HT-4 series II, Genevac Technologies, Suffolk, UK) and the residue (fat content) was weighed. In this analysis one sample from the thylakoid group had to be excluded due to insufficient amount of faecal sample. The analysis therefore includes 17 women in the thylakoid group and 16 women in the control group.

#### Analysis of microbiota

##### DNA-extraction

DNA from faecal samples was isolated and purified using EZ1 DNA Tissue Kit and the robot machine EZ1 Advanced XL (Bacteria card; Qiagen, Hilden, Germany) as previously described [19]. To further disrupt the bacterial cell walls, a step of bead beating for 45 min at 4 °C was added before extraction in the EZ1 Advanced XL. Extracted DNA was stored in 1x TE-buffer at 4 °C until further analyses.

#### Quantitative real-time PCR

The total amount of bacterial 16S ribosomal RNA (rRNA) genes and the amount of 16S rRNA genes of bacteria belonging to the *Clostridium coccoides* group, the *Clostridium leptum* subgroup, *Lactobacillus*, the *Bacteroides fragilis* group, *Enterobacteriaceae* and *Akkermansia muciniphila*-like bacteria were estimated using separate qPCR assays. For all assays, each reaction contained 10 μl 2× Rotor-Gene SYBR Green PCR Master Mix (Qiagen), 0.5 μmol/l of each primer (Table 1), 2 μl of template DNA (extracted as above, 200 ng feaces per 2 μl) and RNAse-free water to the final volume of 20 μl. Samples, standards, and non-template controls were run in triplicate. The thermal cycling was carried out in Rotor-Gene Q (Qiagen) with a programme of 95 °C for 5 min, followed by 40 cycles with denaturation at 95 °C for 5 s, annealing and elongation at 60 °C for 10–30 s. The fluorescent products were detected at the last step of each cycle and melting curve analysis was carried out to ensure specific amplification. Absolute abundance of 16S rRNA genes was calculated based on standard curves using Rotor-Gene Q Series Software 2.1.0 (Qiagen, R2 > 0.996). Detection limit were 10^2^ genes/reaction for the *C. leptum* group, *Lactobacillus*, the *B. fragilis* group, *Enterobacteriaceae* and *A. muciniphila*-like bacteria. Total bacteria and the *Clostridium coccoides* group were detected at 10^3^ 16S rRNA gene copies/reaction. As standard curves, cloned PCR products from *C. coccoides* DSM935, *C. leptum* DSM753, *Lactobacillus plantarum* DSM9843, *B. fragilis* CCUG4856T, *Escherichia coli* CCUG29300T and *A. muciniphila* (a clone confirmed by sequencing) were used. Tenfold dilution series of the target DNA were made in EB buffer (Qiagen). Number of bacteria was expressed as numbers of 16S rRNA gene copies/gram wet weight of faeces.

**Table 1:** Information about the primers used for determination of 16S rRNA genes by qPCR

Target bacterial group	Primer	Sequence (5′-3′)	Amplicon size (bp)	Annealing and elongation time (s)	Reference
Total bacteria	F-tot	GCA GGC CTA ACA CAT GCA AGT C	292	15	[39]
	R-tot	CTG CTG CCT CCC GTA GGA GT			
*Akkermansia muciniphilia*-like bacteria	AM1-F	CAG CAC GTG AAG GTG GGG AC	327	20	[40]
	AM2-R	CCT TGC GGT TGG CTT CAG AT			
*Bacteroides fragilis* group	Bfra-F	ATA GCC TTT CGA AAG RAA GAT	495	30	[41]
	Bfra-R	CCA GTA TCA ACT GCA ATT TTA			
*Clostridium coccoides* group	CcocF	AAA TGA CGG TAC CTG ACT AA	440	20	[41]
	CcocR	CTT TGA GTT TCA TTC TTG CGA A			
*Clostridium leptum* subgroup	CleptF	GCA CAA GCA GTG GAG T	239	20	[42]
	CleptR3	CTT CCT CCG TTT TGT CAA			
*Enterobacteriaecae*	Eco1457-F	CAT TGA CGT TAC CCG CAG AAG AAG C	195	10	[43]
	Eco1652-R	CTC TAC GAG ACT CAA GCT TGC			
*Lactobacillus*	Lact-F	AGC AGT AGG GAA TCT TCC A	341	20	[44]
	Lact-R	CAC CGC TAC ACA TGG AG			[45]

### Thylakoids

The thylakoids (*Appethyl*®) used in the studies were provided by Greenleaf Medical AB (Stockholm, Sweden). The extraction of thylakoids, in this case from spinach leaves, has been described previously [9], with the difference that this powder was obtained after drumdrying. Thylakoids can however be obtained from all green leaves. 100 g of thylakoid-powder contain 41.7 g carbohydrate (of which 38.7 g insoluble fibre), 23.5 g protein and 11.9 g fat.

### Biochemical analyses

The plasma concentration of CCK was measured only in the acute rat study, in the blood-samples taken 30 min after the orogastric bolus dose of Evans blue. CCK was measured using RIA with a highly specific antiserum (92128, detection limit 0.1 pmol/L) according to a previously described method [20].

### Statistical analyses

Statistical data analyses were done using Prism, version 6 (GraphPad software Inc., San Diego, CA, USA). Concerning the rat-experiments, data was controlled for normality using Shapiro-Wilk and KS normality tests, and passed normality for all cases except the acute percentage of Evans migration. Therefore, the Mann Whitney *t*-test was used for this calculation specifically, whereas the Students *t*-test was used for the remaining calculations concerning the rat experiment. Also the descriptive data for the human subjects and the amount of faecal fat content passed normality calculations, and the statistical differences were evaluated with paired Students *t*-test within each treatment group. For the microbiota, statistical evaluations were performed using the non-parametric repeated measures Wilcoxon’s test. A value of *P* ≤ 0.05 was considered significantly different.

## Results

### The effect of thylakoids on gastric emptying and intestinal transit

**Fig. 1:**
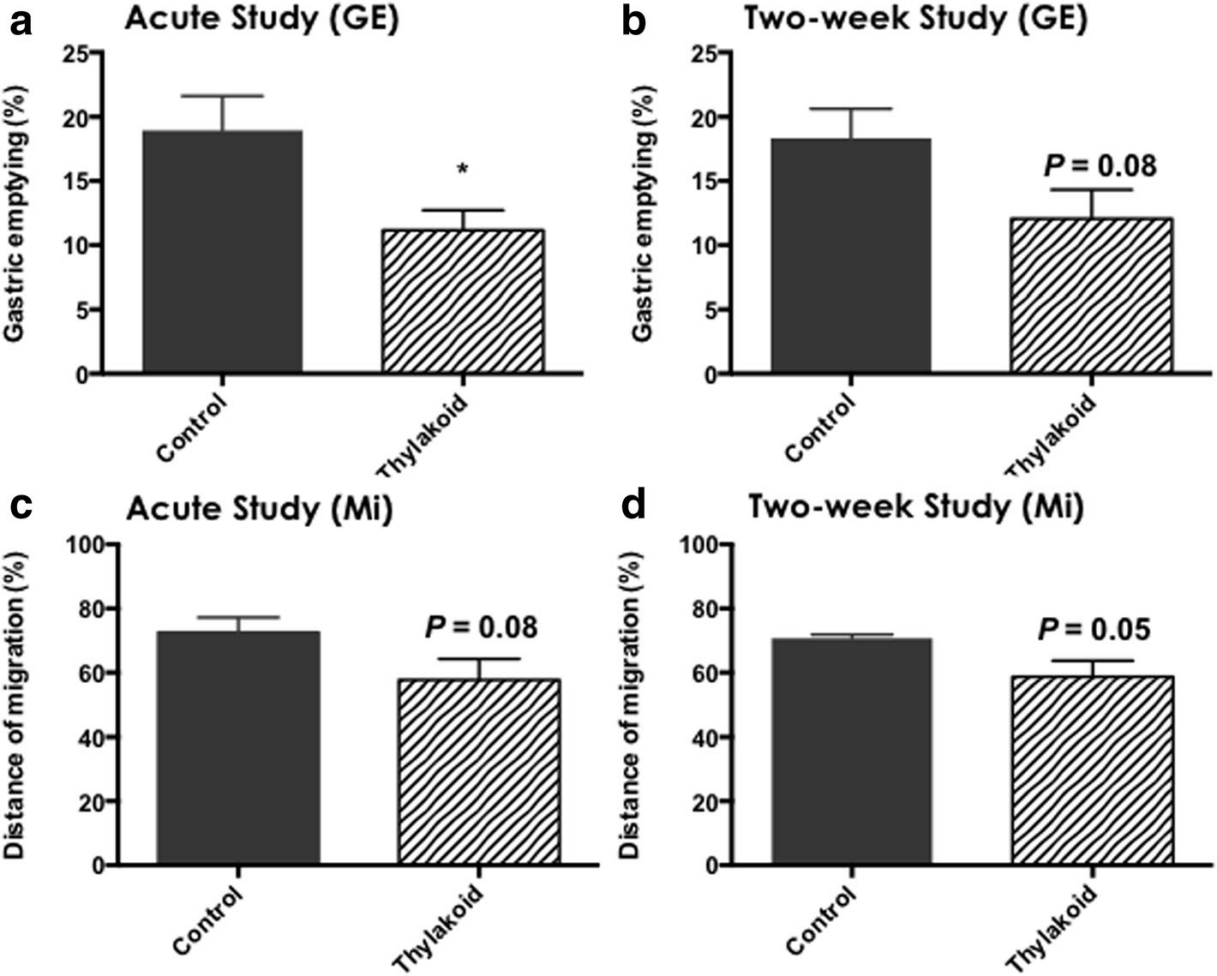
**a**-**d** Decreased gastric emptying (Fig. **a**-**b** (GE), % of total dose of Evans blue) after thylakoid supplementation, both in the acute study where thylakoids in HFD was given as a bolus dose (**a**, control *n* = 6, thylakoid *n* = 8) and after two weeks intake of thylakoids in HFD (**b**, control *n* = 7, thylakoid *n* = 7). Decreased small intestinal motility, measured as the distance of Evans blue migration (Fig. **c**-**d** (Mi), % of total length), both in the acute study where thylakoids in HFD was given as a bolus dose (**c**, control *n* = 6, thylakoid *n* = 8) and after two weeks intake of thylakoids in HFD (**d**, control *n* = 7, thylakoid *n* = 7). Bars represent mean + SEM. Statistical *P*-levels of ≤0.05 was considered to be significant (* = *P* < 0.05)

Rats treated with thylakoids, both acutely and given as a food supplement for two weeks showed decreased gastric emptying and intestinal transit time, as compared to the control rats (Fig. 1a-b (GE)). The gastric emptying was decreased by 41 % in the acute thylakoid experiment (*P* = 0.02), and by 34 % in the two-week thylakoid supplementation study (*P* = 0.08, not significant), compared to the control conditions.

**Fig. 2:**
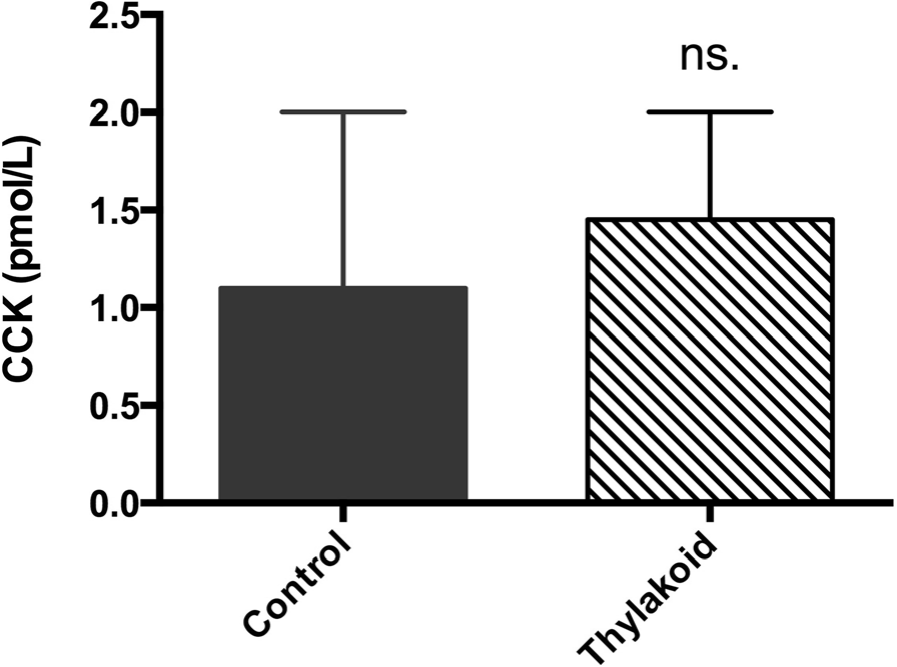
Effect on plasma-concentration of CCK 60 min after a bolus dose of thylakoid supplemented HFD vs. control HFD (control *n* = 6, thylakoid *n* = 8 rats). Bars represent mean + SEM. No statistical difference between thylakoid and control-treated rats was observed

The intestinal transit, measured as the distance the Evans blue had migrated down the intestinal tract during 30 min, was also decreased by thylakoid supplementation, however not significantly (Fig. 1 c-d (Mi)). The migration was decreased after thylakoid supplementation both in the acute thylakoid experiment (*P* = 0.08) and after the two-week thylakoid supplementation study (*P* = 0.05). Thylakoid supplementation had no effect on plasma concentration of CCK (*P* = 0.62) after the bolus doses given in the acute study (Fig. 2). Plasma concentrations were not measured in the two-week diet-supplementation study, since CCK is released in response to nutrients, and in this study we did not control when the rats consumed their food.

### Gastrointestinal effects of daily thylakoid supplementation for three months

**Fig. 3:**
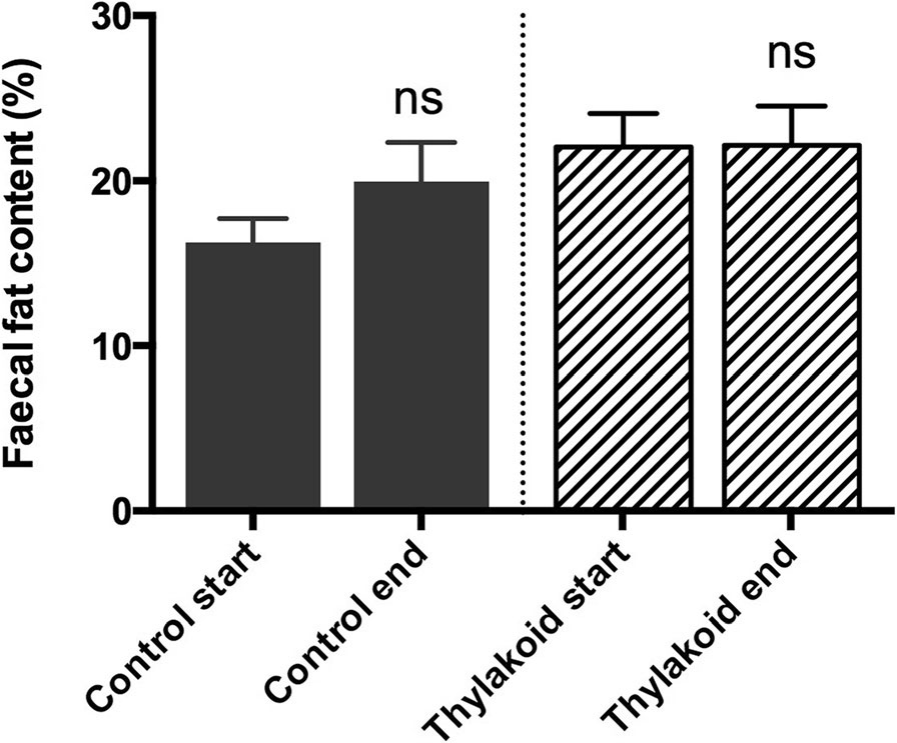
Fat-content in the faecal samples obtained from the overweight women before and after the three-month dietary supplementation period with thylakoids (*n* = 17) or placebo (*n* = 16). No significant difference between before and after the study was found in any of the groups. Bars represent mean + SEM

The fat content in the human faecal samples did not change from before the diet intervention, in either thylakoid or control groups (Fig. 3). The addition of thylakoids did however affect the amount and composition of the gut microbial flora. The quantification of bacteria in the human faecal samples showed a significant increase in the total number of 16S rRNA gene copies after consuming thylakoids for three months compared to before the intervention (*P* = 0.03). In addition, in the thylakoid group the amount of 16S rRNA genes specific for the *B. fragilis* group was significantly higher after (*P* = 0.002), compared to before the intervention, while no significant differences were found for the control group (Table 2). No significant differences were found in either thylakoid or control groups regarding number of 16S rRNA gene copies of *A. muciniphilia*-like bacteria, *C. coccoides* group*, C. leptum* subgroup, *Enterobacteriaceae* or *Lactobacillus* at endpoint compared to baseline (Table 2).

**Table 2:** Concentrations of specific bacterial groups, detected by qPCR, in faeces of volunteers consuming the thylakoid supplement for 3 months compared to volunteers consuming the control supplement. Wilcoxon’s test was used to estimate statistical differences. Medians and interquartile ranges are reported

Bacterial taxa	Control		Control		*P*	Thylakoid		Thylakoid		*P*
	Before intervention		After three months			Before intervention		After three months		
	Log 16S rRNA gene copies/g faeces		Log 16S rRNA gene copies/g faeces			Log 16S rRNA gene copies/g faeces		Log 16S rRNA gene copies/g faeces		
	Median	Range	Median	Range		Median	Range	Median	Range	
Total bacteria	11.0	10.8–11.2	10.8	10.6–11.0	0.464	10.9	10.8–11.1	11.2	11.0–11.4	***0.034***
*Akkermansia muciniphila*-like	8.7	7.8–10.2	9.1	7.7–9.6	0.850	9.9	8.8–10.5	9.6	8.9–10.3	0.744
*Bacteroides fragilis* group	8.7	8.3–9.2	9.2	8.4–9.2	0.252	9.1	8.8–9.6	9.5	9.2–9.9	***0.002***
*Clostridium coccoides* group	10.3	10.2–10.9	10.2	10.0–10.5	0.298	10.5	10.4–10.9	10.8	10.3–11.0	0.325
*Clostridium leptum* subgroup	10.5	10.0–10.6	10.4	9.6–10.6	0.298	10.6	10.1–10.8	10.6	10.3–10.8	0.865
*Enterobacteriaceae*	8.3	7.4–9.0	8.2	6.3–8.7	0.495	7.4	6.6–8.3	7.5	7.0–8.8	0.393
*Lactobacillus*	7.1	6.6–8.5	7.5	6.6–8.3	0.636	7.4	6.8–7.8	7.6	7.0–7.9	0.455

Significant *p*-values are highlighted in bold and italicized

## Discussion

The present study demonstrates that adding thylakoids to the diet decreases gastric emptying and the intestinal transit time, as well as show minor effects on the amount and composition of the gut microbiota. Moreover, the faecal fat content was not affected by thylakoid supplementation for three months.

The decreased gastric emptying and intestinal transit time, however not significant, both in the acute thylakoid-supplementation study and after two weeks of daily thylakoid-intake corroborate previous results of decreased hunger, subjective and objective, obtained after thylakoid supplementation in both animals and humans [9–16, 21, 22]. We have previously shown that thylakoid supplementation to the diet promotes the release of satiety-hormones such as GLP-1 and CCK, and decreases the concentration of the hunger hormone ghrelin [10–12, 15, 16, 23], all of which can be connected to a reduced gastric emptying and decreased intestinal motility. The levels of CCK in the acute study with bolus feeding were not significantly different between the thylakoid and control rats, even though all thylakoid treated rats had slightly increased CCK plasma-concentrations, as compared to the control rats (Fig. 3a). If blood-samples had been taken at more than one time point following the bolus feeding of thylakoid HFD versus control HFD, there would have been better chances of finding significant differences regarding plasma-concentration of CCK. In the two-week supplementation study CCK was not measured since the rats had ad libitum access to food, and CCK is secreted postprandially in response to nutrients present in the intestines [24–26]. Any effects that could have been obtained by measuring CCK would therefore be blunted by the voluntary food intake. In the future, it would be interesting to control food intake in a long-time study to be able to analyse levels of CCK as well as other appetite regulating hormones such as ghrelin, GLP-1 and PYY, and to do this continuously, not as a one-time measurement.

Intake of thylakoids for a longer time period have previously been shown to decrease body weight in humans [12], as well as decrease body weight gain and amount of body fat mass in rodents [9, 10]. Thylakoids have also been found to affect subjective ratings of wanting and liking for food, as well as ratings of hunger, satiety and cravings for palatable food [12–15, 23], all of which can be explained by effects on the appetite-regulating hormones mentioned above. The physiological effect described here of a reduced gastric emptying, together with an increased secretion of hormones such as GLP-1 and CCK, would result in an increased feeling of satiety. This could in turn decrease food intake and thereby promote body weight loss [27–29]. The present study showing a decreased gastric emptying and intestinal transit after thylakoid supplementation, however not in a significant manner, brings a novel mechanistic explanation to the results obtained previously in the thylakoid-appetite research area.

The human faecal samples collected before and after thylakoid or control dietary supplementation for three months, showed that thylakoid-supplementation has no influence on the total intestinal absorption of dietary lipids. Since thylakoids have an affinity for both the lipase/co-lipase complex and to triglycerides [7, 30], it has been discussed whether thylakoids could have the same gastrointestinal side-effects, such as steatorrhea, as have been shown after orlistat-treatment [5]. The amount of faecal fat has previously been shown not to be affected in rats treated with a HFD with or without thylakoid supplementation for 10 days [22]. The present study is however the first study studying faecal fat content after long-term intake of thylakoids in humans. The results of this study, showing no differences in the amount of faecal fat between thylakoid and control, confirms the results from previous studies showing that thylakoids inhibit lipase activity in a reversible fashion [7, 22, 30, 31]. Consequently, we can with certainty say that thylakoid treatment does not cause GI side-effects such as steatorrhea, which are common for other lipase inhibitors [5].

The altered gut microbial composition, however minor, found in the present study, has previously also been showed in a study performed in rats [22]. The gut microbiota has been proposed to be partly responsible for the secretion of appetite regulating hormones such as GLP-1 and PYY [2, 32], since these hormones are secreted from intestinal enterocytes. The gut microbiota also promotes the production of SCFA, which have been proposed to have effects on lipid, glucose and cholesterol metabolism in the host [33, 34]. Indeed, a recent study in rats fed a high-fat diet with or without thylakoids demonstrated that thylakoid supplementation resulted in a reduced respiratory quotient (RQ), indicative of increased fatty acid oxidation [35]. This may have been caused by altered gut microbiota. The oxidation of fatty acids has been found to be increased by SCFA, while the synthesis of lipids is decreased [34]. The results are decreased concentrations of blood lipids and reduced accumulation of lipids, improved glucose and insulin homeostasis, as well as positive effects on the body-weight regulation due to increased secretion of appetite regulating hormones such as GLP-1 and decreased food intake [32, 34, 36–38]. The previous results from thylakoid supplementation in both human and animal interventional studies could thereby, at least partly, be a consequence of an altered gut microbiota. However, this needs to be further elucidated in future studies, including a larger population and a longer study-time. In future studies also the amount of SCFA should be measured, as this is a limitation in the present study.

During the three-month human intervention previously published in Appetite, all subjects reduced their body-weight. However, the present study includes only 34 women out of the original 36 enrolled in the previously study [12]. The differences found in gut microbiota between thylakoid and control group could thereby be caused by body-weight changes, but since the previous study also showed changes in appetite-regulating hormones, we propose it is the other way around, since a change in the gut microbiota have been shown to result in increased secretion of SCFA, PYY and GLP-1, due to colonic fermentation [2, 32]. Previous studies describing the effects of thylakoids have shown decreased amount of cholesterol, increased secretion of satiety hormones, and significant weight-loss [12, 13]. Thereby we propose that also the gut microbiota corroborates in the mechanistic explanations for these results.

## Conclusion

In conclusion, the present study describes a decreased gastric emptying and intestinal transit time after intake of thylakoids in rats. Even though all results are not significantly proven, the results add to the mechanistic explanation to the earlier observed changes in appetite-regulating hormones after thylakoid intake. Whether the delayed gastric emptying is a cause or consequence of the decreased levels of peptides affecting hunger, and elevated levels of peptides affecting satiety and reward in previous studies, remains unanswered. Moreover, the results show that thylakoid consumption does not cause steatorrhea, and that thylakoid intake promotes a minor change in the gut microbiota. The minor change in microbial composition may be caused by weight-loss, but we propose that the weight-loss is the consequence of the change in microflora combined with the change in appetite-regulating hormones as has been shown in earlier studies. Together with previous effects of thylakoid consumption, we propose thylakoids as a promising natural supplement for weight-management, without any known side effects, acting to affect the digestion, metabolism and leading to healthy eating habits.

## Abbreviations

CCK: Cholecystokinin
DCM: Dichloromethane
GE: Gastric emptying
GI: Gastrointestinal
GLP-1: Glucagon-like peptide-1
HFD: High-fat diet
Mi: Gastrointestinal migration
PYY: Peptide tyrosin-tyrosin
RQ: Respiratory quotient
rRNA: Ribosomal RNA
SCFA: Short-chain fatty acids
